# Discovery of Novel Variants on the *CHD7* Gene: A Case Series of CHARGE Syndrome

**DOI:** 10.3389/fgene.2022.852429

**Published:** 2022-07-22

**Authors:** Xiangtao Wu, Liang Chen, Weihong Lu, Shaoru He, Xiaowen Li, Lingling Sun, Longjiang Zhang, Dejuan Wang, Ruigui Zhang, Yumei Liu, Yunxia Sun, Zhichun Feng, Victor Wei Zhang

**Affiliations:** ^1^ The Second School of Clinical Medicine, Southern Medical University, Guangzhou, China; ^2^ Department of Neonatology, Guangdong Provincial People’s Hospital, Guangdong Academy of Medical Sciences, Guangzhou, China; ^3^ Department of Pediatrics of First Affiliated Hospital of Xinxiang Medical University, Xinxiang, China; ^4^ Neonatal Diagnosis and Treatment Center, Children’s Hospital of Chongqing Medical University, National Clinical Research Center for Child Health and Disorders, China International Science and Technology Cooperation Base of Child Development and Critical Disorders, Ministry of Education Key Laboratory of Child Development and Disorders, Chongqing Key Laboratory of Pediatrics, Chongqing, China; ^5^ Shenzhen Children’s Hospital, Shenzhen, China; ^6^ Department of Urology, The Sixth Affiliated Hospital of Sun Yat-sen University, Guangzhou, China; ^7^ Department of Neonatology, Faculty of Pediatrics, Chinese PLA General Hospital, BaYi Children’s Hospital, Seventh Medical Center of Chinese PLA General Hospital, Beijing, China; ^8^ AmCare Genomics Lab, Guangzhou, China

**Keywords:** CHARGE syndrome, infants, respiratory malformations, phenotypes, mutation

## Abstract

**Background:** CHARGE syndrome (CS) is a single-gene genetic disorder with multiple organ malformations caused by a variant of the chromodomain helicase DNA-binding protein 7 (*CHD7*) gene on chromosome 8q12.1. In this study, we aimed to investigate new variants that have emerged in these cases compared with typical CS and the relationship between the genes and phenotypes.

**Methods:** Patients with suspected genetic diseases were subjected to Whole Exome Sequencing (WES) at a genetics laboratory in Guangzhou. The average sequencing coverage depth was >200 ×, and 96% was >20 ×. The variant interpretation was manipulated according to the American College of Medical Genetics (ACMG) guidelines. Molecular data on databases for ClinVar and CHD7 were also collected and collated. We reviewed the currently described *CHD7* variants and analyzed the genetic variation and phenotypic heterogeneity.

**Results:** Data of 12 patients with CS from four hospitals in China were collected. According to gestational age, most of them (8/12) were near-term babies with a lower birth weight than their peers, averaging 2.62 kg. In this study, the most common phenotypes were respiratory tract malformations (11/12), heart malformations (10/12), and central nervous system malformations (9/12). Two fetuses were confirmed to have brain or heart abnormalities during prenatal testing, while 10/12 were found to have abnormalities during prenatal testing. The maximum Acute Physiology and Chronic Health Evaluation (APACHE II) score at admission was 19, and the average was 11.58. Five variants in the *CHD7* gene c.7012C > T (*p*.Q2338*), c.7868delC (*p*.P2623Rfs*16), c.5405-3C > G, c.6936 + 2T > C, and c.8077-2A > G) were novel and were located in exons 33, 36, and introns 25, 32, and 37, respectively. There may be a positive correlation between exon location and phenotype.

**Conclusion:** Five novel variants were discovered. These expanded the mutational spectrum of the *CHD7* gene and the phenotype of CS. There may be a correlation between the new mutation sites and the phenotype, which has some reference value for the evaluation of mutation sites.

## Introduction

CHARGE syndrome (CS) (Online Mendelian Inheritance in Man [OMIM]# 214800) is a rare hereditary congenital anomaly with autosomal dominant transmission caused by the mutation of the chromodomain helicase DNA-binding protein 7 (*CHD7*) gene (OMIM# 608892). It has an incidence of 1/8,500 to 1/15,000 live births worldwide (Janssen et al., 2012). Qin et al. (2020) determined that from nearly 1,000 cases of CS registered in the database. The diagnosis of CS depends on the mutation of the *CHD7* gene and multiple abnormal clinical phenotypes in almost all organs and systems (Hale et al., 2016). The *CHD7* gene was identified and confirmed to be associated with the clinical features of CS and included in the diagnostic criteria, but its pathogenesis remains unclear (Vissers et al., 2004). At present, the research on CS molecular genetic correlation is still progressing. The pathogenic variants of *CHD7* are the key to the diagnosis of CS and were identified as the genetic cause of more than 90% of typical CS patients (Granadillo et al., 2021). The cytogenetic location of *CHD7* is chromosome 8q12.2 and consists of 38 exons. The *CHD7* haploid deficiency is the main cause of CHARGE syndrome (Whittaker et al., 2017). The *CHD7* protein is one of the nine CHD proteins and plays an important regulatory role in embryonic stem cells (Liu et al., 2021). The *CHD7* gene is one of the essential genes for stem cell differentiation and has been found to be involved in embryonic eye development, olfactory nerve stem cells, and semi-circular canal development (Platt et al., 2017). The highly expressed *CHD7* gene regulates histone modification, transcription factor recruitment, and other chromatin remodeling by binding to both active and stable enhancers of ectodermal lineage genes (Goodman and Bonni, 2019).

A retrospective study performed by Biard et al. (2021) discovered that fetal ultrasound and magnetic resonance imaging (MRI) may improve the prenatal diagnosis of internal and external ear abnormalities, posterior nostril atresia, and anencephaly, but these are greatly influenced by changes in the gestation time and prenatal physical signs. Therefore, CS is present from the early fetal stage and indicates the need for prenatal care.

In our study, data of 12 patients with the typical phenotype of CS were collected from four hospitals in different regions of southern China. We identified five new mutation sites and analyzed the genetic variation and phenotypic heterogeneity of all our patients.

## Materials and Methods

### Clinical Data Collection

We conducted a study of 12 children with CS from four hospitals in different regions of Southern China. All patients met the clinical diagnostic criteria of CS updated by Hale et al. (2016). We required all physicians to collect as much clinical information as possible, including gestation period inspection, history of prenatal care, and other data, plus the survival rate during follow-up. All specimens were collected with the informed consent of the family. This study was reviewed and approved by the ethics committee of Guangdong Provincial People’s Hospital.

### Target Capture and Sequencing

Genomic DNA was extracted from peripheral blood using the Solpure Blood DNA kit (Magen Biotechnology) according to the manufacturer’s instructions. The genomic DNA of the patients was then fragmented using a Q800R sonicator (Qsonica) to generate 300–500 bp insert fragments. The paired-end libraries were prepared following the Illumina^®^ library preparation protocol. Custom-designed NimbleGen SeqCap^®^ probes (Roche NimbleGen, Madison, Wisconsin, United States) were used for in-solution hybridization to enrich target sequences. Enriched DNA samples were indexed and sequenced on a NextSeq^®^ 500 sequencer (Illumina^®^, San Diego, California, United States) with 100–150 cycles of single-end reads according to the manufacturer’s protocols.

### Variant Annotation and Interpretation

Primary data came in FASTQ format after the image analysis, and base calling was conducted using the Illumina^®^ pipeline. The data were filtered to generate “clean reads” by removing adapters and low-quality reads (Q20). The average coverage depth of sequencing was >200 ×, and 96% was >20 ×. Sequencing reads were mapped to the reference human genome version hg19 (2009–02 release, http://genome.ucsc.edu/). Nucleotide changes observed in aligned reads were called and reviewed using the NextGENe^®^ software (SoftGenetics, State College, Pennsylvania, United States). In addition to the detection of deleterious mutations and novel single nucleotide variants, the coverage-based algorithm eCNVscan, developed in-house, was used to detect large exonic deletions and duplications. The normalized coverage depth of each exon of a test sample was compared with the mean coverage of the same exon in the reference file to detect copy number variants.

### Data Analysis

Sequence variants were annotated using population and literature databases, including 1000 Genomes, the Single Nucleotide Polymorphism database, the Genome Aggregation Database, ClinVar, the Human Gene Mutation Database, and OMIM. Online software was used to analyze the structure of the protein, predict the conservation and function domains, and perform the multiple sequence alignment. The variant interpretation was manipulated according to the American College of Medical Genetics (ACMG) guidelines (Allyse and Wick, 2017). Molecular data on databases (https://www.chd7.org) for ClinVar and *CHD7* were also collected and collated. Pearson’s correlation analysis was used to analyze the correlation of the exons, phenotypes, and the Acute Physiology and Chronic Health Evaluation (APACHE II) score, where *p* < 0.05 indicated that the result was statistically significant.

## Results

### Clinical Information Analysis

The results of our study are detailed in Table 1 and summarized in Table 2. A total of 12 patients conformed to the diagnostic criteria for CS with *CHD7* gene variants, comprising six male and six female patients, including ten infants. The maximum age was 16 years, and the minimum age was 2 days.

**TABLE1:** Clinical characteristics of 12 patients (N,%) with CS.

N	1	2	3	4	5	6	7	8	9	10	11	12
Age	16 y	6 d	6 m	1 m	2 m	14 d	12 m	2 d	14 d	1 m	8 m	4 y
Gender	M	F	M	M	F	F	M	M	M	F	F	F
Abnormal prenatal care	—	+	+	+	+	+	+	+	+	—	+	+
Gestational age (week)	38	38 + 3	37 + 2	37 + 4	39	40 + 6	35 + 6	36 + 1	39 + 6	39 + 4	36 + 6	36 + 5
Birth weight (kg)	3.1	2.4	2.8	3	2.25	2.95	2.3	2.44	2.95	3.2	2.3	1.7
APACHE Ⅱ score (point)	0	7	8	15	15	16	12	19	18	12	15	2
RDS and pneumonia	—	+	+	+	+	+	+	+	+	+	+	+
Respiratory malformation	—	+	+	+	+	+	+	+	+	—	+	+
Cardiovascular malformation	—	+	—	+	+	+	+	+	+	+	+	+
Nervous system malformation	+	—	+	—	+	—	+	+	+	+	+	+
Developmental delay	+	—	+	—	+	—	+	—	—	+	+	+
External ear malformation	—	+	—	+	+	—	—	+	+	+	+	+
Hearing disorder	+	—	+	—	—	—	+	—	—	—	+	+
Inner ear malformation	+	—	—	—	—	—	—	—	—	—	—	—
Facial asymmetry	—	+	—	—	+	+	—	—	+	—	+	+
Coloboma	—	—	+	+	+	+	+	—	—	—	—	—
Choanal atresia	—	—	—	+	+	—	+	—	—	—	+	
Digestive system abnormality	—	—	—	+	—	+	—	+	—	+	—	—
Urogenital abnormality	—	—	+	+	—	—	+	+	—	—	—	—
Endocrine abnormality	+	—	+	+	—	—	—	—	—	—	+	—
Limb deformities	+	—	—	—	—	—	—	—	—	—	+	—
Electrolyte disorder	—	+	—	+	—	+	—	+	+	+	—	—
Immune abnormality	—	—	+	+	—	—	—	—	—	—	—	—
Allergic history	—	—	—	+	—	+	—	—	—	—	—	—
Nucleotide and amino acid changes	c.253C > T (*p*.Q85*)	c.6018dup (*p*. S2007Ifs*2)	c.7012C > T (*p*.Q2338*)	c.6936 + 2T > C	c.7868delC (*p*.P2623Rfs*16)	c.5405-3C>G	c.7252C > T (*p*.R2418*)	c.8077-2A > G	c.6070C > T (*p*.R2024*)	c.4667dupC (*p*.R1557Kfs*16)	c.780del (*p*.S261Lfs*44)	c.5428C > T (*p*.R1810*)
Type of mutation	Nonsense	Frameshift	Nonsense	Splicing	Frameshift	Intron region	Nonsense	Splicing	Nonsense	Frameshift	Frameshift	Nonsense
Zygote type (heterozygote)	+	+	+	+	+	+	+	+	+	+	+	+
Inheritance (de novo)	+	+	+	+	+	+	+	+	+	+	+	+

M, male; F, female; y, years; d, days; m, months.

**TABLE 2:** Frequency of clinical features of CS patients (N,%).

Hale (2016)	Clinical feature	N	%
Major criteria	Pathogenic CHD7 variant	12	100%
Other phenotype	Respiratory tract malformation	11/12	91.67%
Minor criteria	Heart or esophagus malformation;	10/12	83.33%
Minor criteria	Structural brain anomalies;	9/12	75.00%
Major criteria	Abnormal external, middle or inner ears, including hypoplastic semi-circular canals;	8/12	66.67%
Minor criteria	Developmental delay/intellectual disabilities/autism;	7/12	58.33%
Major criteria	Coloboma;	5/12	41.67%
Other phenotype	Electrolyte disorder	5/12	41.67%
Minor criteria	Cranial nerve dysfunction including hearing loss;	4/12	33.33%
Minor criteria	Hypothalamo-hypophyseal dysfunction (gonadotropin or growth hormone deficiency) and genital anomalies;	4/12	33.33%
Minor criteria	Renal anomalies;	4/12	33.33%
Major criteria	Choanal atresia or cleft lip or palate;	3/12	25.00%
Minor criteria	Dysphagia/feeding difficulties;	2/12	16.67%
Minor criteria	Skeletal/limb anomalies	2/12	16.67%
Other phenotype	Immune abnormality	2/12	16.67%
Other phenotype	Allergic history	2/12	16.67%

2 majors + any number of minor criteria.

Of the 12 patients, there were 11 cases with respiratory malformations and manifestations, including laryngeal or tracheal–bronchial malacia (8/12), neonatal pneumonia (4/12), nostril atresia (1/12), and respiratory distress (8/12). The patients were admitted for respiratory distress syndrome. The main cardiovascular malformations (10/12) were patent ductus arteriosus (5/12), atrioventricular septal defect (4/12), persistent arterial trunk (1/12), and pulmonary hypertension (2/12). There were 9 cases of central nervous system malformations, 1 of which was found on a prenatal MRI. Patient 11 had congenital polydactyly malformations of the left hand, and Patient 7 had a persistent arterial trunk phenotype, which may be a rare phenotype of the *CHD7* gene. Of the 12 patients, 7 patients with developmental delay; 6 patients with an abnormal appearance, manifested as a short neck, right webbed neck, low hairline, micromaxillary deformity, and a skewed mouth angle; 12 patients with internal (1/12) and external (8/12) ear and hearing abnormalities (5/12); and 3 patients with digestive tract abnormalities, including 2 patients with abnormal pharyngeal and palatine arches and another patient with esophageal atresia. There were 3 patients with abnormal vision, including two diseases of the retina and choroid; 2 patients had abnormalities in the urogenital system that showed cryptorchidism and hydronephrosis (Table 1).

Abnormal antenatal examinations were present in ten of the 12 patients. In addition to the four premature infants, there were six low-birth-weight babies, with the lowest being 1.700 kg. The mean birth weight of the 12 patients was 2.62 kg. Abnormalities were found in the low birth weight and premature infants, but most were not examined further. A head MRI of Patient 2 at 28 + 5 weeks’ gestation suggested a slight widening of the left ventricle. Patient 4 had low progesterone in the first trimester and gestational diabetes in the third trimester. Polyhydramnios was detected in Patient 5 in the third trimester. Patient 7 was born prematurely at 35 + 6 weeks after her fetal heart malformation was detected by ultrasound during her mother’s pregnancy without intervention or further examination. In the second trimester, the mother of Patient 8 caught a cold, was not on medication, and no abnormality was found in the late pregnancy examination. Patient 10’s mother tested for prenatal thalassemia. Patient 11’s mother presented with prenatal oligohydramnios. The mothers of patients 6, 9, and 12 had a history of convulsions of hands and feet during pregnancy.

In the patients we studied, there were some variations in the diagnostic criteria. Laryngeal or tracheal-bronchial malacia, lung infection, heart malformation, and central nervous system malformations were the most common phenotypes. The proportion of developmental delay was also higher than the clinical phenotypes in the minor criteria. Electrolyte disturbances were present in five patients, four of whom presented with hypocalcemia. Atopic disorders were present in two patients (Tables 1 and 2). There were four patients with endocrine abnormalities, including hypogonadotropism, hypothyroidism, hypoparathyroidism, and low gonadal hormone secretion. In addition, two patients had an abnormal immune function, one of whom was diagnosed with DiGeorge syndrome.

We assessed the severity of the disease by retrospectively analyzing the APACHE Ⅱ scores at admission. Scores ranged from 0 to 19, with a mean of 11.58. Although only 12 patients were included in the study, we found that the younger the age, the higher the critical illness score, indicating the severity of the disease. Patients 4–11 were in a critical condition with APACHE Ⅱ scores >12. They were treated with non-invasive or invasive ventilators at admission. However, Patients 8–10 did not respond well to the treatments, and the guardian finally gave up treatment and discharged them. The other patients are still alive, but most have abnormal respiratory tract development, leading to recurrent lung infections.

### Mutation Analysis

**FIGURE 1:**
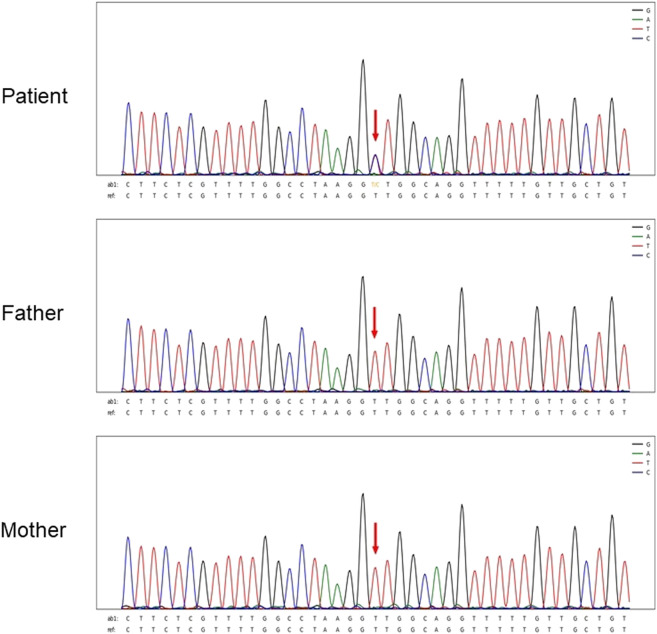
Sanger sequencing results for Patient 4. The chromatographs show that heterozygous mutation of the CHD7 gene can be detected, a mutant c.6936 + 2T > C was splicing mutation, and the patient’s parents did not have this mutation. c.6936+2T > C mutation is indicated by arrows.

**FIGURE 2:**
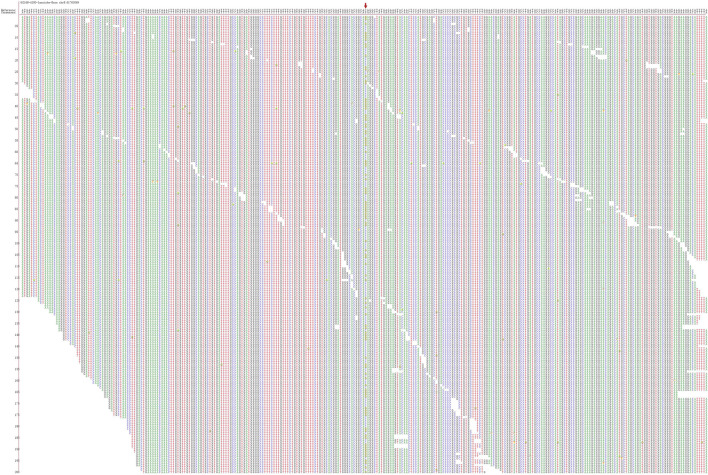
Second-generation sequencing maps for Patient 6.

**FIGURE 3:**
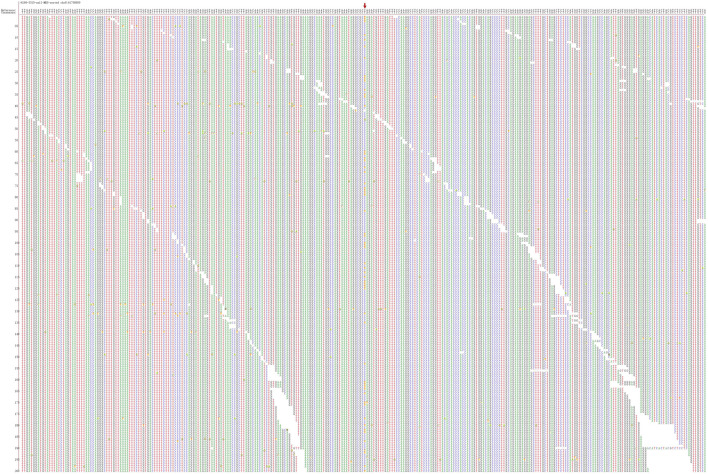
Second-generation sequencing maps for Patient 3 shows CHD7 heterozygous mutation–positive, but c.7012C > T is a nonsense mutation.

**FIGURE 4:**
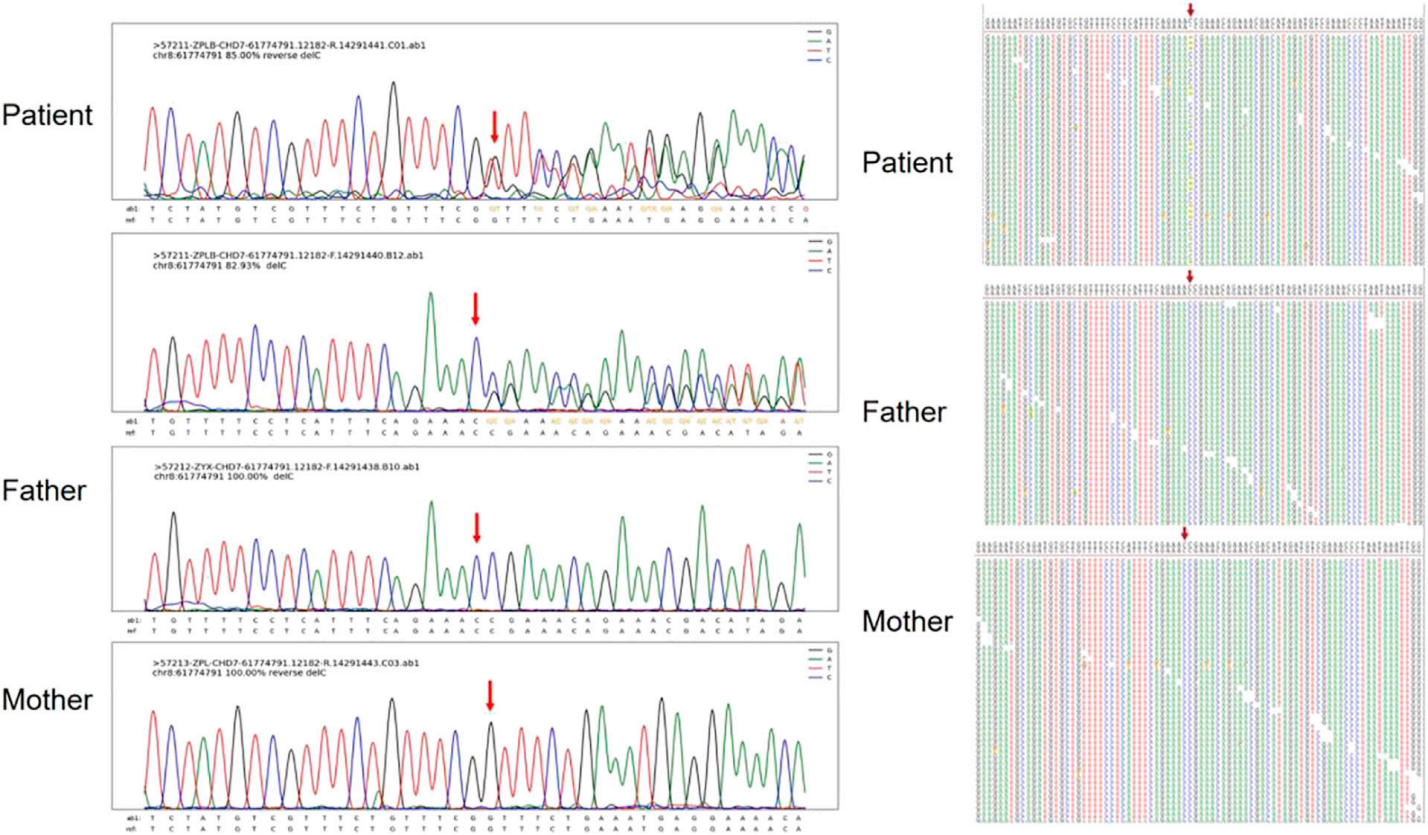
Sanger sequencing results and maps for Patient 5. The chromatographs show that heterozygous mutation of the CHD7 gene can be changed, a mutant c.7868delC was frameshift mutation, and the patient’s parents did not have this mutation. c.7868delC mutation is indicated by arrows.

Sanger sequencing after genomic DNA was extracted from 12 patients, and these variants interpretation was manipulated according to the American College of Medical Genetics (ACMG) guidelines. All patients were found to be CHD7 heterozygous mutation positive and these variants were *de novo*. There were two cases of splicing mutation: c.6936+2T > C (Figure 1) and c.8077-2A > G. The two splicing mutations and c.5405-3C > G were located in introns (Table 3; Figure 2). There were five cases of nonsense mutation: c.253C > T (*p*.Q85*), c.7012C > T (*p*.Q2338*) (Figure 3), c.7252C > T (*p*.R2418*), c.6070C > T (*p*.R2024*), and c.5428C > T (*p*.R1810*). There were four cases of frameshift mutation: c.6018dup (*p*.S2007Ifs*2), c.7868delC (*p*.P2623Rfs*16) (Figure 4), c.4667dupC (*p*.R1557Kfs*16), and c.780del (*p*.S261Lfs*44). Except for c.253C > T (*p*.Q85*) (PubMed reference number [PMID] 21258681), c.6018dup (*p*.S2007Ifs*2) (PMID 20884005), c.7252C > T (*p*.R2418*) (PMID 21258681), c.6070C > T (*p*.R2024*) (PMID 15300250), c.4667dupC (*p*.R1557Kfs*16) (PMID:23024289), c.780del (*p*.S261Lfs*44) (PMID 21258681), and c.5428C > T (*p*.R1810*) (PMID 16400610), the others were confirmed as novel mutations of the *CHD7* gene.

**TABLE 3:** Gene and mutation types of CHD7 in 12 CS patients (N,%).

Patient No	Gene	Zygote type	Inheritance	HG19 position	Nucleotide and amino acid changes	Type of mutation	Reported
1	CHD7	Heterozygote	De novo	chr8:61654244	c.253C > T (p.Q85*)	Nonsense	Yes
2	CHD7	Heterozygote	De novo	chr8:61765175	c.6018dup (p. S2007Ifs*2)	Frameshift	Yes
3	CHD7	Heterozygote	De novo	chr8:61768609	c.7012C > T(p.Q2338*)	Nonsense	No
4	CHD7	Heterozygote	De novo	chr8:61767084	c.6936 + 2T > C	Splicing/ Intron region	No
5	CHD7	Heterozygote	De novo	chr8:61774791	c.7868delC (p.P2623Rfs*16)	Frameshift	No
6	CHD7	Heterozygote	De novo	chr8:61763049	c.5405-3C > G	Intron region	No
7	CHD7	Heterozygote	De novo	chr8:61769091	c.7252C > T (p.R2418*)	Nonsense	Yes
8	CHD7	Heterozygote	De novo	chr8:61777573	c.8077-2A > G	Splicing/ Intron region	No
9	CHD7	Heterozygote	De novo	chr8:61765232	c.6070C > T (p.R2024*)	Nonsense	Yes
10	CHD7	Heterozygote	De novo	chr8:61754427	c.4667dupC (p.R1557Kfs*16)	Frameshift	No
11	CHD7	Heterozygote	De novo	chr8: 61654768	c.780del (p.S261Lfs*44)	Frameshift	Yes
12	CHD7	Heterozygote	De novo	chr8: 61763075	c.5428C > T(p.Arg1810Ter)	Nonsense	Yes

Alamut was used to predict the effect of each mutation on protein translation. The splicing and protein function predicted by Alamut may have been affected by c.6936 + 2T > C and c.8077-2A > G, while c.5405-3c > G was less likely to affect the splicing.

**FIGURE 5:**
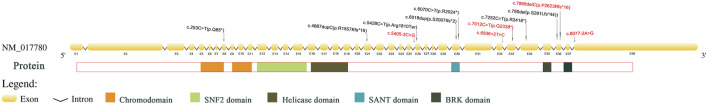
Pathogenic CHD7 variants (top) of CHARGE syndrome in the patients we studied according to the chromosomal location. The sites that are color-coded are those novel mutations. Below the CHD7 gene is a protein domain that corresponds to the location of the gene that encodes it.

Figure 1 shows Sanger sequencing results for Patient 4. The chromatographs show that a heterozygous mutation of the *CHD7* gene was detected: a mutant c.6936 + 2T > C was a splicing mutation of intron, and the patient’s parents did not have this mutation. We mapped the 12 variants into the structure of the *CHD7* gene (Figure 5 and Table 4). On the transcriptional protein domain, one variant was located in the chromodomain, two were located in the SANT domain, and the others were distributed in the remaining exon region. Exons 33, 36 and Introns 25, 32, 37 had one new mutation each, and we found two mutation sites in exon 36.

**TABLE 4:** CHD7 phenotype and location of mutation site in 12 CS patients.

Location of mutation	HG19 position	Nucleotide and amino acid changes	Protein domain	Number of phenotypes (n)	ACMG	Reported (PMID)	N
Exon 8	chr8:61654244	c.253C>T (p.Q85*)	Chromodomain	7	1	Yes/21158681	1
Exon 21	chr8:61754427	c.4667dupC (p.R1557Kfs*16)	ATP-binding site	5	2	Yes/32625235	10
Intron 25	chr8:61763049	c.5405-3C > G	Unknown	8	1	No	6
Exon 26	chr8:61763075	c.5428C > T (p.R1810*)	Unknown	8	1	Yes/16400610	12
Exon 30	chr8:61765175	c.6018dup (p.S2007Ifs*2)	SANT	6	1	Yes/20884005	2
Exon 30	chr8:61765232	c.6070C > T (p.R2024*)	SANT	7	1	Yes/15300250	9
Intron 32	chr8:61767084	c.6936 + 2T>C	Unknown	12	2	No	4
Exon 33	chr8:61768609	c.7012C > T (p.Q2338*)	Unknown	9	2	No	3
Exon 34	chr8:61769091	c.7252C > T (p.R2418*)	Unknown	9	2	Yes/16155193	7
Exon 34	chr8:61654768	c.780del (p.S261Lfs*44)	Unknown	11	1	Yes/21158681	11
Exon 36	chr8:61774791	c.7868delC (p.P2623Rfs*16)	Unknown	9	1	No	5
Intron 37	chr8:61777573	c.8077-2A > G	Unknown	8	1	No	8

ACMG 1: pathogenicity.

ACMG 2: possible pathogenicity.

**FIGURE 6:**
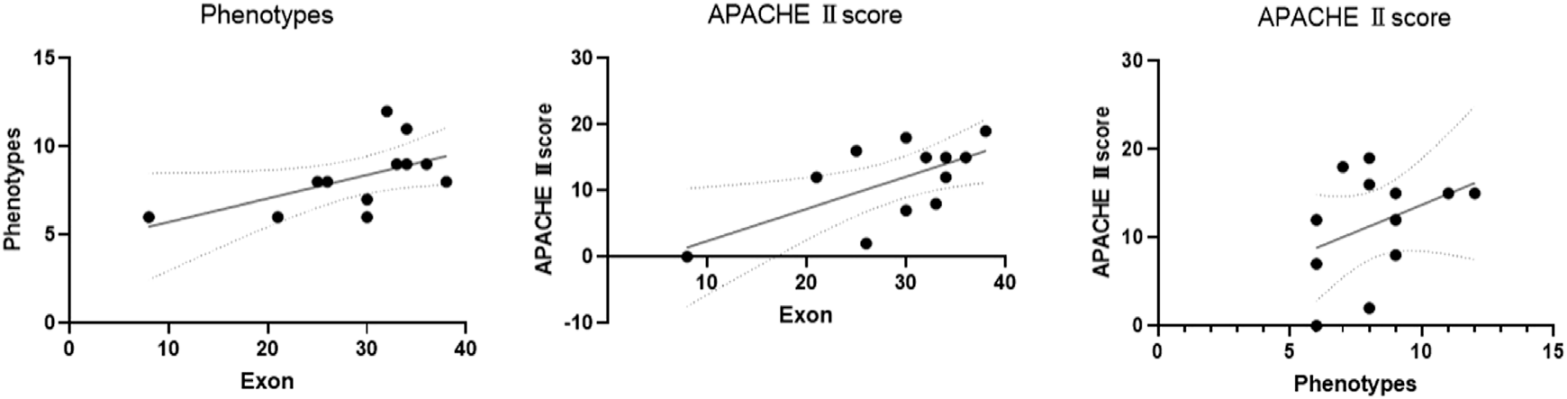
Pearson correlation analysis was used to analyze the correlation of the exons, phenotypes, and APACHE Ⅱ score. The scatter plot showed that exon location was positively correlated with phenotype number and APACHE Ⅱ score (exon vs. phenotypes: R = 0.58, *p* = 0.0487; exon vs. APACHE Ⅱ score: R = 0.65, *p* = 0.0233). There was also a positive correlation between the number of phenotypes and the APACHE Ⅱ score (R = 0.38, *p* = 0.219). ACMG 2: possible pathogenicity.

The scatter plot showed that exon location was positively correlated with phenotypes and the APACHE Ⅱ score (exon site vs. phenotypes: R = 0.58, *p* = 0.0487; exon site vs. APACHE Ⅱ score: R = 0.65, *p* = 0.0233). There was also a positive correlation between the number of phenotypes and the APACHE Ⅱ score (R = 0.38, *p* = 0.219) (Table 4; Figure 6).

According to data analysis from the ClinVar and CHD7 gene databases, a total of 1,806 variants are *CHD7* gene mutations, where 1,054/1,806 are pathogenic/likely pathogenic variants that mainly consisted of frameshift (462/1,054), nonsense (327/1,054), missense (155/1,054), and splicing (120/1,054) mutations, while missense (613/768) mutations had the highest probability of benign/likely benign or uncertain significance.

## Discussion

Hall (1979) and Hittner et al. (1979) first described a series of congenital malformations of this syndrome in 1979. In 1981, Pagon (Whittaker et al., 2017) used the acronym “CHARGE” (ocular coloboma [C], heart malformations [H], atresia of the choanae [A], retardation of growth [R], genital hypoplasia [G], and ear abnormalities [E]) to describe the clinical manifestations of this multiple malformation, which gave people a systematic understanding of CHARGE syndrome. In recent years, the diagnosis of CHARGE syndrome has increased due to the rapid development of whole-exome sequencing, which was identified as important in guiding health care policy development (Fung et al., 2020). *De novo* mutations made up 87% of autosomal dominant diseases (Yang et al., 2014). We identified 12 patients with CS due to *CHD7* mutations, of which 5 were novel mutations consistent with *de novo*.

Genetic analysis of our patients found that all 12 variants were *de novo*. Our findings broaden the spectrum of clinical features associated with pathogenic variants. Of the 12 cases that we investigated, there were five (5/12) cases of nonsense, four (4/12) cases of frameshift, two (2/12) cases of splicing, and one (1/12) case of intron. The pathogenicity of various mutation types was close to that reported by Qin et al. (2020). Through the analysis of the data in the ClinVar and *CHD7* databases, it was found that frameshift, nonsense, and splicing mutations were the most likely to be pathogenic/likely pathogenic, and the number of frameshift mutations was the largest. Missense mutations were of uncertain significance and benign/likely benign. This is similar to a study in France, which found that missense mutations may be associated with a milder phenotype (Legendre et al., 2017).

In this study, five reported mutations are new and pathogenic. Except for c.5405-3C > G located in intron, the other five are in exons. Predictions using the Alamut software suggested that the intron variant seems unlikely to have had an impact on protein translation, but the phenotype is typical. The transcriptional effects of this intron mutation need to be further studied. On the transcriptional protein domain for these 12 variants, one variant was in the chromodomain, two were in the SANT domain, and the others were distributed in the remaining exon region. Exons 33, 36 and Introns 25, 32, 37 had one new mutation each, and we found two mutation sites in exon 36. By comparison with the protein functional domain, the relationship between these variants and the phenotype is still unclear. Therefore, Pearson’s correlation analysis was used to analyze the correlation of the exons, phenotypes, and APACHE Ⅱ scores. The exon sites were positively correlated with the APACHE Ⅱ scores (R = 0.65), indicating significance (*p* = 0.0233). There was also a positive correlation between exon position and phenotypes (R = 0.58), but the difference was not significant (*p* = 0.0487). The correlation coefficient between the number of phenotypes and the APACHE Ⅱ score was smaller than the previous two results, and the difference was also not significant (R = 0.38, *p* = 0.219). Our finding shows that there may be a correlation between the severity of phenotype with the location of exon mutation. Legendre et al. (2017) and (Shotelersuk et al., 2020) found no significant correlation between phenotype and genotype.

C. 253C > T (*p*.Q85*), C. 780del (*p*.S261Lfs*44), C. 7252C > T (*p*.R2418*), and C. 5428C > T (P.ARg1810ter) were mainly reported in molecular studies of gene mutations, without specific descriptions of clinical phenotypes (Koenighofer et al., 2015; Zaki et al., 2018; Shotelersuk et al., 2020; Lee et al., 2021). C. 6018DUP (*p*.S2007Ifs*2) was reported in a study related to endocrine and olfactory, but there was no more phenotypic description (Lin et al., 2020). In this study, the phenotype of C.4667dupC (P. r1557KFS *16) was basically consistent with previous reports. The difference was mainly due to the fact that endocrine detection was not performed in this case, and the patient gave up treatment and was discharged from the hospital due to a critical condition at the age of 1 month. C.6070C > T (*p*.R2024*) in this case, the new phenotype is tracheomalacia presenting with respiratory malformation, postnatal RDS and recurrent pneumonia. Compared with previous reports, new phenotypes of C.7252C > T mutations were neurological malformation, respiratory malformation with RDS, urinary tract malformation, and coloboma (Koenighofer et al., 2015). In addition, by analyzing these novel mutations with the functional domain of the *CHD7* protein, it was found that the exons of these mutations were not in the functional domain of the known *CHD7* protein. This suggests that phenotypes may not be significantly related to specific functional domains (Gug et al., 2020; Qin et al., 2020). Therefore, whether the gene expression and chromatin structure are correct may be the pathogenesis of CS caused by the *CHD7* mutation. The *CHD7* gene may be regulated by epigenetic and signaling pathways that function as chromatin remodeling factors and may directly or indirectly affect ectodermal lineage genes (Platt et al., 2017; Legendre et al., 2018; Goodman and Bonni, 2019). Abnormal antenatal examinations were present in ten of the 12 patients. Prompt prenatal care can detect early signs of most developmental abnormalities, provide more informed medical management, and allow for a precise determination of reproductive risks. However, there are few high-quality studies about prenatal diagnosis, and it is challenging to carry out. In this study, the most common phenotypes were respiratory tract malformation (11/12), heart malformation (10/12), and central nervous system malformations (9/12). We assessed the severity of the disease by retrospectively analyzing the APACHE Ⅱ score at admission, where the score ranged from 0 to 19 with a mean of 11.58. Although only 12 patients were included in the study, we found that the younger the age, the higher the critical illness score, indicating the severity of the disease.

## Conclusion

This study collected typical patients with CHARGE syndrome in Southern China, identified five novel mutation sites, expanded the mutation spectrum of the *CHD7* gene and the phenotype of CS, and provided guidance for clinical diagnosis and genetic counseling. Prenatal examination is very helpful for the early diagnosis of CS, and respiratory malformation may be one of the important phenotypes of its severity. There may be a correlation between new mutation sites and phenotypes, and the evaluation of mutation sites has a certain reference value.

## Data Availability

The original contributions presented in the study are included in the article/Supplementary Material; further inquiries can be directed to the corresponding author.
